# Combining family history and alcohol screening measures to identify emerging adults at risk of not being in education, employment, or training (NEET)

**DOI:** 10.1007/s00127-025-02904-5

**Published:** 2025-04-28

**Authors:** Julie E. Brummer, Kirsten Søndergaard Frederiksen, Katherine J. Karriker-Jaffe, Katie N. Kim, Karen G. Chartier

**Affiliations:** 1 Centre for Alcohol and Drug Research, Aarhus BSS, Aarhus University, Campus Emdrup, Tuborgvej 164, Building A, 2nd Floor, 2400 Copenhagen NV, Denmark; 2 Centre for Alcohol and Drug Research, Aarhus BSS, Aarhus University, Bartholins Allé 10, Building 1322, 8000 Aarhus C, Denmark; 3 Center for Health Behavior & Implementation Science, RTI International, 2150 Shattuck Avenue, Suite 800, Berkeley, CA 94704 USA; 4 School of Social Work, Virginia Commonwealth University, 1000 Floyd Avenue, Richmond, VA USA; 5 School of Social Work and Virginia Institute for Psychiatric and Behavioral Genetics, Virginia Commonwealth University, 1000 Floyd Avenue, Richmond, VA USA

**Keywords:** AUDIT-C, NEET, Family history, Substance use, Alcohol screening, Emerging adults

## Abstract

**Purpose:**

Being outside of the labor and education system during young adulthood, a status termed not in education, employment, or training (NEET), is a risk factor for later social and health outcomes. This study examined whether parental substance use (PSU) moderates the relationship between personal alcohol consumption and NEET. Such information may inform screening practices.

**Methods:**

Participants included 2,940 respondents (15–25-year-olds) to a 2014 Danish national survey. In this historical cohort study, survey data were linked with register data on respondents’ parents and follow-up register data on respondents’ educational/employment status (2015–2018). The Alcohol Use Disorders Identification Test-Consumption (AUDIT-C) identified respondents with hazardous drinking. PSU was measured using survey and register data. Our outcome identified those who were NEET during 1 + years follow-up. Analyses included gender stratified multivariable logistic regressions.

**Results:**

Survey-based PSU was associated with higher odds of NEET (OR = 1.67, *p* <.001). An AUDIT-C + by survey PSU interaction was statistically significant for females (OR = 2.70, *p* =.007): AUDIT-C + females with survey PSU had higher odds of NEET than AUDIT-C + females without survey PSU. Results were similar using register PSU. For both genders, other parental factors (unemployment and psychiatric problems) were also associated with higher odds of NEET.

**Conclusion:**

Using family history (including unemployment, psychiatric problems, and substance use) may enhance screening to identify emerging adults at risk of adverse social outcomes. Survey- and register-based PSU were similarly associated with NEET, suggesting that asking emerging adults about their parents’ substance use may be as informative as more objective measures of family risk.

**Supplementary Information:**

The online version contains supplementary material available at 10.1007/s00127-025-02904-5.

## Introduction

Young people’s detachment from the educational system and labor market – a status that has been termed not in education, employment, or training (NEET) [1]– has an impact both at the individual and societal levels [2–4]. Youth detachment from the labor market has been shown to have a “scarring” effect [4], meaning that being NEET may reduce future socioeconomic opportunities, putting young people at risk of further exclusion from the labor market later in life [5, 6]. The long-lasting effects of being NEET in emerging adulthood have been observed in poor economic outcomes 20 years later [6] and even at ages close to retirement [7]. In addition to being an important social determinant of health for young people, being NEET also has societal consequences [8] in terms of the effects on economic growth, unemployment, and welfare payments [9]. In the European Union, 11.2% of young people (aged 15–29) were considered to be NEET in 2023, with the highest proportion in Romania (19.3%) and the lowest in the Netherlands (4.8%) [10]. In Denmark, where the present study was conducted, approximately 8.6% of young people were estimated to be NEET [10].

The far-reaching effects of being NEET during emerging adulthood point to the importance of identifying risk factors and screening for being NEET. While little research has focused on the development and validation of NEET-specific screening instruments, the Risk of NEET Indicators (RONI) is implemented in some educational institutions in the UK [11, 12]. RONI systems are adapted by local authorities, and risk factors may include, for example, excessive school absences, health problems, low family income, homelessness, low self-confidence, minority ethnicity, and substance use or heavy alcohol use [11, 12].

Although research on screening instruments has been limited, a substantial number of studies have focused on factors associated with NEET status. Indicators such as parental unemployment and low parental income have been shown to increase the likelihood of young people being NEET [13]. Parental substance use problems (PSU) have also been associated with being NEET in emerging adulthood [14], poor school performance, attendance, and youth unemployment [15–18], although the evidence on whether a young person’s own substance use predicts being NEET is mixed [13, 19]. The interplay between personal consumption and PSU may offer some insight into these findings. PSU is a risk factor for offspring drug and alcohol problems and use disorders [20, 21], as well as other short- and long-term health and psychosocial consequences [22, 23]. Heavy parental alcohol consumption has also been shown to be associated with a heavier drinking trajectory among emerging adults [24]. PSU may increase NEET risk not only through its socio-economic and psychological effects, but also by influencing youth drinking behaviors. In PSU-affected households, young people may experience disrupted household routines and lower parental supervision, which can lead to earlier initiation of alcohol use and higher-risk drinking patterns [25]. Such behaviors further out have been linked to poorer educational and employment outcomes [25], reinforcing cycles of social and economic exclusion. A study of individuals who had been in treatment for a substance use disorder found a relationship between PSU and being NEET two years after treatment enrollment [26]. It remains unexplored whether combining indicators of personal consumption with family history of substance use may improve identification of at-risk emerging adults, which is the focus of our analysis.

Information on family history of both mental and physical health conditions is readily accessible to researchers in Nordic countries due to the long history of national medical registers and use of unique personal and family identifiers that make it possible to link registers [27]. However, in Denmark, such data can only be used for scientific and statistical purposes [28], and register data are not accessible to practitioners in clinical settings. Outside of Nordic countries, administrative data on family health history may be accessible for research purposes in the records of private insurance providers if family members are covered by the same company [29]. However, as family members’ health records are generally not available to clinicians, one aim of the present study was to examine whether family history ascertained using administrative records and family history based on youth reports similarly identify at-risk individuals.

Building on prior research, the present paper examines how indicators of personal alcohol consumption and family history of substance use problems jointly may improve identification of being NEET. We assess results using complementary objective register-based measures and subjective youth-report survey-based measures of PSU. Self-reports may indicate personal experiences of parental drinking behaviors and attitudes, family conflict, and early access to alcohol that also predict later problem drinking and social problems among youth themselves [30, 31], and a self-report may capture PSU which is less severe than that identified by their parents’ register data from sources such as hospital and crime records [32]. However, self-reports can be biased due to factors such as loyalty to parents or feelings of shame [33, 34]. By including both survey- and register-based indicators, we can take into consideration the strengths and limitations of both measures and explore differences in interactions with personal alcohol consumption on NEET status of young adults. Prior research has shown that there are differences between males and females related to both alcohol screening and the relationship between an individual’s family history and their own alcohol use outcomes [35–40]. As such, we use stratified models to understand these complex relationships to better identify young men and women at elevated risk for being NEET.

## Methods

In this retrospective cohort study, young Danish adults were followed in national registers for four years (2015–2018). The study population consisted of respondents to the 2014 Danish National YouthMap Survey, which had a participation rate of 55.5% (3,064 of the random sample of 5,520 15–25-year-olds identified from the Danish Civil Registration System by Statistics Denmark). Participants completed the survey through a web-based questionnaire or a telephone interview. Survey questions addressed well-being, psychosocial problems and strengths, and substance use (for further details, see [41, 42]). The sample for the present study includes 2,940 survey respondents who could be matched to a national register on personal labor market affiliation, had not emigrated during the four-year, register-based follow-up period, and provided sufficient information to calculate AUDIT-C scores.

### Outcome

*NEET.* Respondents’ education and employment status during the four years following the survey was identified using a national register that records labor market affiliation during the majority of the year based on information on income, education, workplace sector, and social benefits [43, 44]. For each of the four follow-up years, participants were categorized as being NEET if they were unemployed, temporarily out of the labor force (e.g., receiving medical or parental leave benefits), retired, receiving social assistance (e.g., cash benefits), or categorized as “other.” Alternatively, participants were considered to be in education, employment, or training if they were self-employed, employed, or enrolled in an education program. For regression analyses, participants’ NEET status during the follow-up was dichotomized (≥ 1 year NEET vs. 0 years NEET), as NEET is not a common outcome and any years disconnected from education or employment could be considered problematic [45]. Participants’ NEET status at baseline (in 2014) was included as a covariate in sensitivity analyses.

## Main exposures

### The Alcohol Use Disorders Identification Test-Consumption (AUDIT-C)

The Alcohol Use Disorders Identification Test-Consumption (AUDIT-C) is a validated [36, 46, 47] and widely-recommended [48] instrument for identifying individuals who consume alcohol at hazardous or harmful levels. The AUDIT-C is an abbreviated version of the 10-question AUDIT [49, 50], consisting of the first three questions measuring the frequency of alcohol use, usual quantity of alcohol consumed per drinking day, and frequency of binge drinking. This screening tool has been tested in diverse adult populations [51–53] and has been found to be a valid screening instrument among adolescents and emerging adults drawn from psychiatric clinics, primary care, and student populations aged 12–19 years [54], 14–18 years [55], and college-aged [56, 57]. The predictive validity of the AUDIT-C has been demonstrated by several recent studies, with scores above the cutoff associated with an increased risk for future alcohol problems [58], violence victimization [59], and hospital admission for alcohol-related conditions [60]. At a given level of consumption, blood alcohol concentration is higher in females than males, and females experience alcohol-related diseases at lower levels of alcohol use than males [61, 62]. Accordingly, sex differences have been found in the optimal cutoffs applied to alcohol screening tools, with a lower score recommended for identifying excessive drinking for females than for males [35, 36].

The AUDIT-C was collected in the 2014 survey; however, as response options differed slightly from those in the original AUDIT-C, we used the method for recoding responses applied in a subsequent Danish YouthMap survey [63]. The AUDIT-C was scored on a scale of 0–12, with a maximum of four points per question. An AUDIT-C score of 0 indicates that the respondent drank no more than one time per month. The present study used a cutoff of five for males and four for females (AUDIT-C+) [56, 64, 65].

### History of PSU

History of PSU was identified using survey- and register-based indicators, as detailed in prior studies [42, 66]. Survey-based PSU was defined as an affirmative response to a survey question concerning whether or not their parent(s) had ever misused alcohol or drugs. Parents of respondents were identified using a family relations register [67]. Register-based PSU was defined as one or more of the following substance-related register entries for a parent during the period between 1989 (or the year the register was established, if it was after 1989) and 2015: (a) a hospital admission for a substance-related diagnosis (other than tobacco-related diagnoses) [68, 69]; (b) a conviction for a substance-related crime [70]; (c) a substance use treatment episode [71, 72]; (d) prescription of medication for the treatment of substance use disorders (excluding nicotine) [73]; or (e) a substance-related cause of death [74]. There are differences between females and males in risk factors for alcohol problems [37], with some evidence suggesting familial history of alcohol dependence might be a stronger risk factor for females than males [38], although results have varied by the populations studied [39, 40].

### Covariates

Additional relevant demographic covariates included respondent sex, respondent age, parents’ highest level of education, parental history of psychiatric problems, and parental long-term unemployment [18, 75, 76]. Sex (male or female) was based on survey self-report. Age at the time of the survey was treated as a dichotomous variable (15–18 vs. 19–25) to reflect distinct stages of young adulthood (during vs. after high school). Parents’ highest level of education when the child was 15 was trichotomized (compulsory education, upper secondary, and higher education) and was identified using the education register. Parental psychiatric problems were defined as any admission to a psychiatric hospital during the period 1989–2015 (except for substance-related admissions), identified using the Psychiatric Central Research Register. Parental long-term unemployment was defined as three consecutive years, or more than three non-consecutive years, of unemployment or receipt of social benefits during the respondent’s childhood (birth through age 15), identified using registers on parents’ labor market affiliation.

### Statistical analysis

We first conducted descriptive analyses for the whole sample and for females and males separately. Multivariable logistic regression models were then used to examine associations of AUDIT-C + and PSU with NEET. After these main effects were analyzed, interactions of AUDIT-C + and PSU on NEET were assessed. Interactions that did not achieve statistical significance (*p* <.05) in the sex-stratified models were dropped for parsimony; the final, reduced models for females and males are presented. In sensitivity analyses, we included baseline NEET as a covariate, and we also (separately) assessed whether an interaction between AUDIT-C + and family history on NEET status extended to parental psychiatric problems.

Post-hoc analyses were conducted to explore the extent to which an association between AUDIT-C score and NEET status may be affected by abstention status. We used a chi-square test to compare the proportions of abstainers (defined as those with a score of 0 on the AUDIT-C), non-abstaining AUDIT-C- respondents (scores below the cutoff but above 0), and AUDIT-C+ respondents (scores above the cutoff) who were NEET during the follow-up. To assess whether abstainers might be socially isolated, we also compared these three groups in terms of the proportion with fewer than four close friends (from survey data) using a chi-square test.

All models included respondents with complete data on study variables. All analyses were conducted using STATA v.17 [77] using weights created by Statistics Denmark to reflect the age, sex, family structure, parental education, and origin (i.e., Danish origin, immigrant or descendant) of the Danish population.

## Results

### Description of study population

Total sample and sex-stratified descriptive statistics are presented in Table 1. In terms of respondents’ alcohol use, 30.5% of both females and males were AUDIT-C+. Among all respondents, 29.0% were abstainers (AUDIT-C score of 0). Approximately 19.5% of respondents had a parent with a substance use problem (14.0% for survey-based PSU and 13.1% for register-based PSU). Similarly, 12.7% of respondents had a parent who had a psychiatric problem, with just 4.9% of respondents having a parent with both substance use and psychiatric problems. Parental long-term unemployment was experienced by 25.1% of respondents. Most respondents were employed or undertaking an education/training at baseline (90.5%) and throughout follow-up (75.3%). Broad reasons for NEET are presented in Table 2. Females were significantly more likely than males to report survey PSU (*p =.03)*, but we did not find evidence that males and females differed significantly on any of the other assessed measures, including register-based PSU.

**Table 1:** Study population characteristics by sex (*N* = 2,940)

	Weighted (%)			
	Overall	Females	Males	*p* ^c^
Total		48.98	51.02	
Age group (years)				
15–18	35.48	35.12	35.82	0.694
19–25	64.52	64.88	64.18	
AUDIT-C > cutoff	30.48	30.66	30.31	0.841
History of PSU				
Survey-based PSU	13.95	15.51	12.46	**0.030**
Register-based PSU	13.08	12.74	13.41	0.620
Parents’ highest level of education				0.358
Compulsory	15.26	16.03	14.52	
Upper secondary	49.55	50.09	49.03	
Higher education	35.19	33.88	36.45	
Parental history of psychiatric problems^a^	12.65	11.64	13.63	0.127
Parental history of long-term unemployment^b^	25.13	24.25	25.95	0.338
NEET at baseline	9.54	9.56	9.53	0.985
Years of NEET during follow-up				
0	75.31	73.89	76.67	0.166
1	11.27	12.35	10.23	
2	5.43	6.00	4.88	
3	3.24	3.54	2.94	
4	4.76	4.22	5.28	

Abbreviations: AUDIT-C, Alcohol Use Disorders Identification Test-Consumption; NEET, not in education, employment, or training

^a^ Parental psychiatric problems defined based on either parent’s admission(s) to a psychiatric hospital for non-substance use problems

^b^ 89 missing

^c^*p*-value for test of sex differences; statistically significant results are **bold**

**Table 2:** Distribution of reasons for being NEET

NEET indicator (based on main source of income)	% (number of years)
Registered as unemployed at least half of the year	10.4 (140)
Received sick leave, parental leave, or other leave benefits	13.4 (180)
Received disability pension	6.0 (81)
Received cash benefits	22.8 (307)
Other^a^	47.4 (637)

^a^ Not studying, had little or no connection to the labor market (earned income < 58,600 DKK in 2016 prices (approx. 8,700 USD), and received minimal income from unemployment and cash benefits (under 58,600 DKK in 2016 prices) [78]

### Full sample analyses

For the full sample, we did not find evidence of a significant interaction effect for survey- or register-based PSU with AUDIT-C+ (see Models 3 and 4 in the Online resource). In the main effects model that included survey-based PSU, an AUDIT-C + score was associated with lower odds of being NEET (OR = 0.74; 95%CI = 0.60–0.92), while PSU (OR = 1.67; 95%CI = 1.29–2.18) was associated with increased odds of being NEET (Table 3). Other parental factors were similarly associated with an increased risk of being NEET (unemployment: OR = 1.73; 95%CI = 1.38–2.15; psychiatric problems: OR = 1.57; 95%CI = 1.20–2.06). In the main effects model that included the register-based measure, PSU was significantly associated with increased odds of being NEET (OR = 1.33; 95%CI = 1.01–1.74), and the other variables had a similar relationship to being NEET as in the model that included survey-based PSU.

**Table 3:** Multivariable logistic regression models: main effects for the full study population

	Adjusted Main Effect Model (*N* = 2,851)					
	Model 1 Survey PSU				Model 2 Register PSU	
	Adjusted OR [95%CI]	*p*			Adjusted OR [95%CI]	*p*
AUDIT-C > Cutoff						
Yes	0.74 [0.60, 0.92]	0.005**			0.75 [0.61, 0.93]	0.008**
Survey-based PSU						
Yes	1.67 [1.29, 2.18]	< 0.001***			--	
Register-based PSU						
Yes	--				1.33 [1.01, 1.74]	0.039*
Gender						
Female (vs. Male)	1.14 [0.95, 1.38]	0.167			1.16 [0.96, 1.40]	0.117
Age group						
19–25 (vs. 15–18)	1.30 [1.07, 1.58]	0.007**			1.35 [1.11, 1.63]	0.003**
Parental psychiatric problems^a^						
Yes	1.57 [1.20, 2.06]	0.001**			1.59 [1.21, 2.08]	0.001**
Parental long-term unemployment^b^						
Yes	1.73 [1.38, 2.15]	< 0.001***			1.77 [1.42, 2.20]	< 0.001***
Parents’ highest level of education						
Upper secondary (vs. Compulsory)	0.70 [0.50, 0.98]	0.039*			0.70 [0.50, 0.98]	0.036*
Higher education (vs. Compulsory)	0.72 [0.51, 1.01]	0.060			0.71 [0.50, 1.00]	0.053

Abbreviations: PSU, parental substance use problems; AUDIT-C, Alcohol Use Disorders Identification Test-Consumption; NEET, not in education, employment, or training

^a^ Parental psychiatric problems is based on registry data and defined as either parent’s admission(s) to a psychiatric hospital for non-substance use problems

^b^ Parental long-term unemployment is based on registry data and defined as 3 consecutive years or more than 3 non-consecutive years of either parent’s unemployment or receipt of social welfare benefits during respondent’s childhood

Statistically significant effects are **bold**; * *p* <.05; ** *p* <.01; *** *p* <.001

### Sex-stratified analyses

**Fig. 1:**
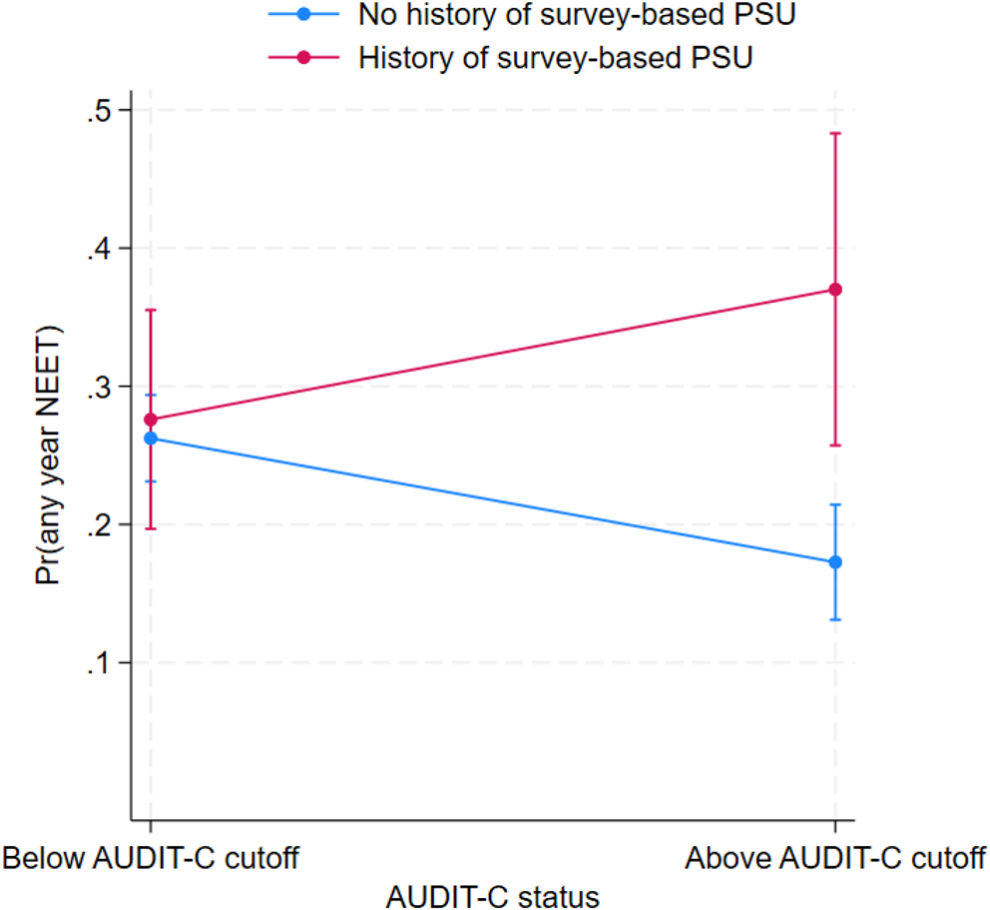
Predictive margins estimated for females and showing the interaction effect between AUDIT-C + status and survey-based PSU in association with being NEET

For females, but not males, we found statistically significant multiplicative interactions between both measures of PSU and AUDIT-C + on odds of being NEET (Table 4). Figure 1 shows the predictive margins for the interaction between AUDIT-C + and survey-based PSU. The figure indicates that females who were AUDIT-C + with a history of PSU had a higher risk of being NEET than females who were AUDIT-C + without PSU. As we did not find evidence of an interaction effect among males, we present results from main effects models (Table 4). AUDIT-C + was not significantly associated with being NEET. However, males with survey-based PSU (OR = 1.88; 95%CI = 1.28–2.75), as well as males whose parent(s) had psychiatric problems (OR = 1.63; 95%CI = 1.14–2.34) or long-term unemployment (OR = 1.91; 95%CI = 1.40–2.61) were significantly more likely to have been NEET during follow-up. Register-based PSU was not significantly associated with being NEET.

**Table 4:** Multivariable logistic regressions: final models for females and males

	**Interaction Models in Females (*N*** **= 1,416)**				**Main Effect Models in Males (*N*** **= 1,435)**			
	Model 5 **AUDIT x Survey PSU**		Model 6 AUDIT x Register PSU		Model 7 Survey PSU		Model 8 Register PSU	
	Adjusted OR [95%CI]	*P*	Adjusted OR [95%CI]	*p*	Adjusted OR [95%CI]	*p*	Adjusted OR [95%CI]	*p*
AUDIT-C > cutoff								
Yes	0.58 [0.41, 0.81]	0.001**	0.59 [0.42, 0.82]	0.002**	0.77 [0.57, 1.05]	0.096	0.79 [0.58, 1.07]	0.124
Survey-based PSU								
Yes	1.07 [0.69, 1.68]	0.755	--		1.88 [1.28, 2.75]	0.001**	--	
Register-based PSU								
Yes	--		0.98 [0.61, 1.57]	0.930	--		1.30 [0.89, 1.89]	0.175
Age group								
19–25 (vs. 15–18)	1.54 [1.17, 2.03]	0.002**	1.59 [1.21, 2.10]	0.001**	1.13 [0.86, 1.49]	0.385	1.16 [0.88, 1.52]	0.302
Parental psychiatric problems^a^								
Yes	1.51 [1.01, 2.26]	0.045*	1.43 [0.95, 2.15]	0.083	1.63 [1.14, 2.34]	0.008**	1.67 [1.16, 2.39]	0.006**
Parental long-term unemployment^b^								
Yes	1.57 [1.15, 2.15]	0.005**	1.57 [1.14, 2.16]	0.005**	1.91 [1.40, 2.61]	< 0.001***	1.97 [1.46, 2.68]	< 0.001***
Parents’ highest level of education								
Upper secondary (vs Compulsory)	0.72 [0.44, 1.15]	0.168	0.73 [0.45, 1.18]	0.197	0.71 [0.44, 1.14]	0.154	0.68 [0.42, 1.09]	0.107
Higher education (vs Compulsory)	0.63 [0.38, 1.03]	0.066	0.64 [0.39, 1.05]	0.078	0.84 [0.51, 1.36]	0.476	0.80 [0.49, 1.30]	0.367
Interactions								
AUDIT-C x Survey PSU	2.70 [1.31, 5.56]	0.007**	--		--		--	
AUDIT-C x Register PSU	--		3.03 [1.41, 6.51]	0.004**	--		--	

Abbreviations: PSU, parental substance use problems; AUDIT-C, Alcohol Use Disorders Identification Test-Consumption; NEET, not in education, employment, or training

^a^ Parental psychiatric problems is based on registry data and defined as either parent’s admission(s) to a psychiatric hospital for non-substance use problems

^b^ Parental long-term unemployment is based on registry data and defined as 3 consecutive years or more than 3 non-consecutive years of either parent’s unemployment or receipt of social welfare benefits during respondent’s childhood

Statistically significant effects are **bold**; * *p* <.05; ** *p* <.01; *** *p* <.001

### Sensitivity analyses

Including baseline NEET status as a covariate did not substantially alter the interaction effects identified for females (survey-based PSU x AUDIT-C+: OR = 2.58; 95%CI = 1.18–5.63; register-based PSU x AUDIT-C+: OR = 3.21; 95%CI = 1.39–7.42). We also found a significant interaction between parental psychiatric problems and AUDIT-C + for females in models accounting for either survey- or register-based PSU (parent psychiatric x AUDIT-C+: OR = 3.11; 95%CI = 1.36–7.10 and OR = 2.98, 95%CI = 1.31–6.80, respectively).

### Post-hoc analyses

NEET status during follow-up was more prevalent among abstainers (34.7%) than among non-abstaining respondents who scored below the AUDIT-C cutoff (21.8%) and among respondents who were AUDIT-C+ (19.3%) (*p* <.001). Abstainers were more likely to have fewer than four close friends (38.9%), compared to drinkers who scored below the AUDIT-C cutoff (29.8%) and compared to respondents who were AUDIT-C+ (16.5%) (*p* <.001).

### Discussion

With the overall aim of investigating whether adding information on family history of substance use to an alcohol screening tool improves the estimation of being NEET, we examined whether PSU during childhood and adolescence moderates the relationship between self-reported risky alcohol use and subsequent poor education and employment outcomes during emerging adulthood. In the full sample, we found that both emerging adults’ own excessive alcohol consumption as well as their parents’ history of problematic substance use predicted their future education and employment status. While drinking at levels above the AUDIT-C cutoff was associated with *lower* odds of being out of the labor market and educational system during follow-up, PSU was associated with *higher* odds of subsequent classification as being NEET. We did not find evidence of an interaction between these variables in the full sample; however, when females were assessed separately, PSU modified the relationship between AUDIT-C + status and being NEET, such that for those who had experienced PSU, scores above the AUDIT-C cutoff were associated with a significantly increased risk of being NEET. This result suggests that, for female emerging adults, it is important to consider the interplay between their own alcohol consumption and parental history when identifying those at increased risk for poor social outcomes. For male emerging adults, the main effects models suggest that parental history was more strongly associated with risk of being NEET than the young person’s own alcohol consumption.

In the present study, nearly one-third of respondents scored above the AUDIT-C cutoff. This is consistent with international surveys, which have demonstrated the relatively low prevalence of abstinence and high prevalence of intoxication among Danish young people [79–81]. For instance, results of a recent European School Survey Project on Alcohol and Other Drugs (ESPAD) survey found 74% of Danish participants had consumed alcohol in the past month and 40% had been intoxicated, compared with the ESPAD student averages of 47% and 13%, respectively [80]. In terms of screenings, a 2014 study using AUDIT cutoffs to define risk status found that a minority of Danish university students qualified as drinking at a “low-risk” level [82].

We found a negative association between AUDIT-C + status and being NEET. This contrasts with some prior international research showing that alcohol-related indicators, such as early alcohol use [83] and adolescent heavy drinking [84, 85], are associated with adverse educational and employment outcomes. However, some previous work has called into question the importance of alcohol as a risk factor for such outcomes [86–88]. A recent systematic review and meta-analysis [19] found mixed results for an association between alcohol use and NEET status in young people, and in the two studies in which alcohol use was measured prior to NEET status, there was no evidence of a significant association (OR 0.80, 95%CI 0.48–1.34).

The results of the present study may be explained at least in part by a positive relationship between drinking and social inclusion among Danish youth. In Denmark, the legal minimum age for purchase of beer and wine is 16, which is lower than in most other countries [89], and risky drinking among youth is prevalent [90]. In heavy-drinking youth cultures, young people who abstain from alcohol or consume alcohol at a low level may feel stigmatized, lonelier, and less socially accepted than their peers [91, 92]. A national youth survey in Denmark found that the relationship between weekly alcohol consumption and poor well-being was U-shaped, with a significantly higher risk of self-reported poor well-being among those who never or occasionally drank and among those who drank very heavily (≥ 22 units/week), compared to those who drank 1–7 units per week [93].

As suggested by qualitative studies, the Danish context of low abstention rates and widespread heavy drinking both contributes to and results from a youth culture centered on alcohol, in which there is an increased risk of social exclusion for those who do not drink [94–96]. The results of our post-hoc analyses support this explanation, as the abstaining respondents in our study had smaller social networks. The results of our study are also in line with findings from an evaluation of alcohol use among Danish higher education students showing that students who abstained at the beginning of their studies were less socially integrated, although the highest dropout rates during the first year were among both the heaviest drinkers and those who abstained [97]. Prior work on the relationship between adolescent mental and physical health with educational and employment outcomes suggest that social exclusion is an important mediating variable [98, 99]. The results of our study offer some evidence that abstainers, in so far as this indicator is correlated with social exclusion, may need to be considered differently when assessing risk for future social problems.

Another possible explanation for the negative association between AUDIT-C status and being NEET is that although we controlled for parental education level and long-term unemployment, there may have been some residual confounding related to socio-economic status, with young people from families with lower incomes drinking less frequently [100], possibly due to having less disposable income to spend on alcohol, but having an increased risk of being NEET [101].

While the relationship between emerging adult drinking and employment and educational outcomes is somewhat disputed in the literature, our findings showing that PSU predicts NEET status are in line with the large body of evidence demonstrating that children and young people who have experienced PSU are at increased risk of poor education outcomes [18, 102–104], unemployment [15], and being NEET, specifically [14]. In our study, females were more likely than males to report PSU when surveyed. Similar sex differences have been observed in other Danish youth surveys [105] as well as in other Nordic countries [106, 107]. Related sex differences extend to alcohol’s harms to others research as well, which has shown that females are more likely than males to report experiencing alcohol-related harm from drinkers who are known to them [108]. These types of self-report data, however, cannot distinguish whether females experience more adverse consequences, whether they are more likely to attribute such consequences to a family member’s drinking, or whether they are more willing to report such events and attributions in a survey. A study by Roy et al. [109] suggested that females have a lower threshold than males for identifying relatives’ substance use as problematic, although a similar study by Rice et al. [110] found no such sex difference. We identified significant interaction effects for AUDIT-C + with both survey- and register-based PSU for females, but no interaction effects with either measure of PSU in males. Prior research on this population found some female subgroups were at higher risk for social and health harms than males when exposed to PSU in childhood, particularly in families where the parents did not live together for the entire 15 years of childhood [25].

Our findings suggest that, for females, screening for elevated risk of adverse outcomes such as being NEET will be enhanced by considering both drinking behaviors and family history. The consistent interaction effect for females using both register-based and youth-reported measures of PSU indicates that self-reported family history, which is relatively simple to obtain in a clinical setting, can be a valuable contribution to screening. Health systems increasingly have web-based portals where patients can enter their own family history data as well as modules in electronic health records for providers to review and update during visits [111, 112], and such information could be used to supplement alcohol screening using tools such as the AUDIT-C. However, substance use disorders are particularly stigmatizing, even compared with other mental disorders [113, 114]. Thus, young people may be reluctant to report that they have a parent with substance use problems if their answers are non-anonymized, such as in a clinical setting where screening may take place. However, we found a similar interaction between AUDIT-C + and non-substance related parental psychiatric problems. Thus, our results suggest it may be sufficient to ask young females about family psychiatric history in general, rather than about specific conditions.

Future research should utilize other study designs to evaluate the effectiveness of integrating family history items into screening tools. Raising awareness about family health history could be a personal motivator for changing risky health behaviors when combined with a brief intervention. Family history information is routinely collected for the BASICS assessment (Brief Alcohol Screening and Intervention for College Students), and it is returned to participants as a part of their personalized feedback report [115]. There is evidence from studies of both alcohol and cannabis use that brief interventions are more efficacious at reducing substance use in young people who have a family history of alcohol or drug problems [116, 117], including one study testing a female-specific motivational enhancement intervention [118].

### Limitations

This study was conducted in a setting where a high proportion of the young adult population drinks alcohol frequently and heavily [79–82] and where alcohol consumption is deeply embedded in the culture. Findings might not generalize to populations where young people have different patterns of drinking, abstention is more prevalent, and/or participation in drinking occasions is less entwined with social inclusion. Our study population consisting of a cohort of emerging adults in 2014 allowed us to have a relatively long period of follow-up; however, there is a risk there may be some cohort effects. It would therefore be interesting for future studies to replicate these analyses using later cohorts and longer follow-up periods. Because we aimed to assess whether existing screening tools and commonly applied cut-points could be improved by incorporating information about problematic parental substance use, we based our cut-points on prior research. However, these may not represent the optimal cut-points for a population of Danish young people, and future studies could include diagnostic interviews or consumption diaries as the gold standard to generate receiver operating characteristic (ROC) curves and determine optimal AUDIT-C cutoffs. Further, there also are some limitations to our choice of outcome. NEET status is a heterogeneous indicator that captures different reasons for being outside the educational system and workforce (including taking a sabbatical before beginning higher education or taking parental leave after a child’s birth), not all of which indicate the same level of vulnerability and risk of long-term social or economic exclusion [119, 120], particularly in the Nordic countries, where extended paid parental leave is common. Thus, it may not be the case that all instances of NEET indicate an adverse outcome.

## Conclusions

Results of our study indicate that PSU plays a role in the association between individual alcohol use and risk of being NEET. Our findings provide preliminary support for the utility of supplementing information on personal alcohol consumption with family history of substance use problems to identify female emerging adults at increased risk of future negative outcomes. This study further strengthens the evidence that adverse childhood experiences, such as parental long-term unemployment, psychiatric problems, and substance use, are significant independent predictors of being NEET in young adulthood for both males and females. Reports of PSU were consistently associated with NEET, suggesting that family history items administered in a clinical setting may be as effective as more objective measures for identifying people at increased risk of long-term social consequences.

## Electronic supplementary material

Below is the link to the electronic supplementary material.

Supplementary Material 1

## Data Availability

Danish register data are only available via an application to Statistics Denmark and an affiliation with a Danish-based institution.
