# Supplementary material for: Combining family history and alcohol screening measures to identify emerging adults at risk of not being in education, employment, or training (NEET)
Source: Soc Psychiatry Psychiatr Epidemiol. 2025 Apr 28;60(11):2563–75. doi: 10.1007/s00127-025-02904-5 (PMC12572071; doi:10.1007/s00127-025-02904-5)
Supplement: Supplementary file 1 — Supplementary Material 1 [file 127_2025_2904_MOESM1_ESM.docx]

**Table 1** Multivariable logistic regression models: Interaction effects for the full study population

|  |  |  | **Adjusted Interaction Model (*N*=2,851)** | | | | |
| --- | --- | --- | --- | --- | --- | --- | --- |
|  |  |  | **Model 3**  **AUDIT-C x Survey PSU** | |  | **Model 4**  **AUDIT-C x Register PSU** | |
|  |  |  | **Adjusted OR [95%CI]** | ***p*** |  | **Adjusted OR [95%CI]** | ***p*** |
| AUDIT-C > cutoff | |  |  |  |  |  |  |
|  | Yes |  | 0.68 [0.54, 0.86] | **0.001**** |  | 0.70 [0.55, 0.88] | **0.002**** |
| Survey-based PSU | |  |  |  |  |  |  |
|  | Yes |  | 1.47 [1.08, 2.01] | **0.016*** |  | -- |  |
| Register-based PSU | |  |  |  |  |  |  |
| Yes | |  | -- |  |  | 1.17 [0.85, 1.60] | 0.344 |
| Gender | |  |  |  |  |  |  |
|  | Female (vs Male) |  | 1.14 [0.94, 1.37] | 0.180 |  | 1.15 [0.96, 1.39] | 0.135 |
| Age group | |  |  |  |  |  |  |
|  | 19-26 (vs 15-18) |  | 1.31 [1.08, 1.60] | **0.006**** |  | 1.36 [1.12, 1.65] | **0.002**** |
| Parental psychiatric problems^a^ | |  |  |  |  |  |  |
|  | Yes |  | 1.58 [1.21, 2.06] | **0.001**** |  | 1.58 [1.21, 2.06] | **0.001**** |
| Parental long-term unemployment^b^ | |  |  |  |  |  |  |
|  | Yes |  | 1.72 [1.38, 2.14] | **< 0.001***** |  | 1.76 [1.42, 2.19] | **< 0.001***** |
| Parents’ highest level of education | |  |  |  |  |  |  |
| Upper secondary (vs Compulsory) | |  | 0.70 [0.50, 0.98] | **0.038*** |  | 0.69 [0.50, 0.97] | **0.033*** |
| Higher education (vs Compulsory) | |  | 0.71 [0.51, 1.01] | 0.056 |  | 0.71 [0.50, 1.00] | **0.048*** |
| Interactions | |  |  |  |  |  |  |
|  | AUDIT-C x Survey PSU |  | 1.51 [0.88, 2.60] | 0.139 |  | -- |  |
|  | AUDIT-C x Register PSU |  | -- |  |  | 1.60 [0.91, 2.79] | 0.100 |

Abbreviations: PSU, parental substance use problems; AUDIT-C, Alcohol Use Disorders Identification Test-Consumption; NEET, not in education, employment, or training.

^a^ Parental psychiatric problems is based on registry data and defined as either parent's admission(s) to a psychiatric hospital for non-substance use problems.

^b^ Parental long-term unemployment is based on registry data and defined as 3 consecutive years or more than 3 non-consecutive years of either parent's unemployment or receipt of social welfare benefits during respondent's childhood.

Statistically significant effects are **bold**; * *p* < .05; ** *p* < .01; *** *p* < .001
